# Does thermoregulatory behavior maximize reproductive fitness of natural isolates of *Caenorhabditis elegans?*

**DOI:** 10.1186/1471-2148-11-157

**Published:** 2011-06-06

**Authors:** Jennifer L Anderson, Lori Albergotti, Barbara Ellebracht, Raymond B Huey, Patrick C Phillips

**Affiliations:** 1 Center for Ecology and Evolutionary Biology, University of Oregon, Eugene, OR 97402, USA; 2 Department of Biology, University of Texas at Arlington, Arlington, TX 76019, USA; 3 Department of Biology, University of Washington, Seattle, WA 98195, USA; 4 Department of Zoology, University of Florida, Gainesville, FL 32611, USA

## Abstract

**Background:**

A central premise of physiological ecology is that an animal's preferred body temperature should correspond closely with the temperature maximizing performance and Darwinian fitness. Testing this co-adaptational hypothesis has been problematic for several reasons. First, reproductive fitness is the appropriate measure, but is difficult to measure in most animals. Second, no single fitness measure applies to all demographic situations, complicating interpretations. Here we test the co-adaptation hypothesis by studying an organism (*Caenorhabditis elegans*) in which both fitness and thermal preference can be reliably measured.

**Results:**

We find that natural isolates of *C. elegans *display a range of mean thermal preferences and also vary in their thermal sensitivities for fitness. Hot-seeking isolates CB4854 and CB4857 prefer temperatures that favor population growth rate (*r*), whereas the cold-seeking isolate CB4856 prefers temperatures that favor Lifetime Reproductive Success (LRS).

**Conclusions:**

Correlations between fitness and thermal preference in natural isolates of *C. elegans *are driven primarily by isolate-specific differences in thermal preference. If these differences are the result of natural selection, then this suggests that the appropriate measure of fitness for use in evolutionary ecology studies might differ even within species, depending on the unique ecological and evolutionary history of each population.

## Background

How organisms respond to environmental variation and change, both in the short term via mechanisms such as behavior modulation and in the long term via evolutionary changes in features such as the pattern of reproductive allocation, remains a central question in ecology and evolutionary biology. Temperature variation in particular has profound effects on the physiology, ecology, and fitness of ectotherms [1-5]. Extreme temperatures are injurious and potentially lethal, but even temperatures within lethal limits dramatically change performance and ultimately Darwinian fitness [3,6,7]. Even so, most ectotherms are not at the mercy of the thermal environment but rely on behavioral adjustments ([8], but see [9]) to regulate body temperatures at usually narrow, species-specific levels. These behaviors are thought to be adaptive in two complementary ways. (i) They help animals avoid extreme temperatures that can be damaging or lethal [10,11]. (ii) They increase the time spent at physiologically optimal temperatures, thus enhancing overall physiological performance and survival in nature [10,12-17]. For these reasons, thermal preferences are widely assumed to maximize Darwinian fitness [18].

**Figure 1:**
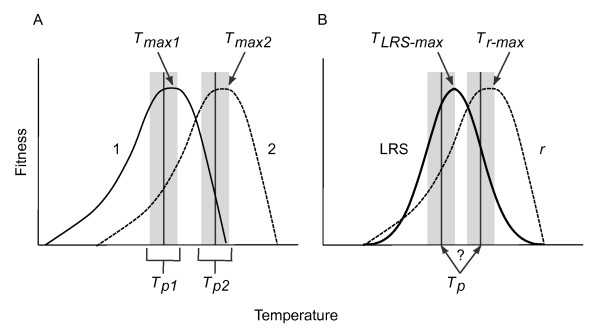
**Expected coevolution of thermal preference and fitness**. Under the coadaptation hypothesis, organisms are expected to prefer a narrow range of temperatures (*T_p_*) that corresponds to, but is slightly lower than [6], body temperatures that maximize their fitness (*T_max_*). A) If this hypothesis is valid, populations with relatively high *T*_p _will have relatively high *T*_max_, as depicted here. B) However, the thermal sensitivity of Lifetime Reproductive Success (LRS) is generally shifted to a lower temperature than that maximizing the intrinsic rate of increase (*r*) (see [3]); thus, the *T*_p _of a given population can correspond with *T_LRS-max _*or *T_r-max_*.

More formally, a long-standing adaptive hypothesis proposes that behavior and physiology co-evolve, such that thermal preferences should be centered at or near temperatures that maximize fitness (Figure 1A) [12,18-21]. These temperatures are often called "preferred body temperatures" (*T_p_*) and are estimated in the laboratory via thermal gradients [22]. Previous approaches to testing this classical coadaptation hypothesis reveal that preferred body temperatures often correlate with optimal temperatures for various performance traits, though generally not 1:1 [6]. In any case, these tests are not ideal because they use performance (*e.g*. traits such as sprint speed and digestion [2,18,19,23,24]) as a proxy for fitness. The reason for using fitness proxies is obvious: estimating fitness at multiple temperatures is extremely difficult with most ectotherms. Nevertheless, although performance influences fitness, it is not fitness [25-27]. Furthermore, although interspecific comparisons are important, intraspecific level tests of this hypothesis are especially informative because such comparisons are less likely to be confounded by other inter-species differences and potentially more likely to be the result of adaptation to the local environment (*i.e*. be tied to fitness differences between populations). Thus, an advance in testing the co-adaptational hypothesis would involve examining the relationship between thermal preference and the temperatures maximizing Darwinian fitness in a species that shows intraspecific variation in thermal preference and in which the thermal dependence of fitness can be readily and reliably estimated.

According to the co-adaptational hypothesis, population mean fitness would be maximized if all individuals in a population thermoregulate perfectly: if so, individuals will prefer and experience only the narrow range of temperatures that results in the highest fitness (but see [6]). However, organisms - either in laboratory thermal gradients or in nature - at least temporarily occupy a range of temperatures. From the coadaptation hypothesis, it naturally follows that such imperfect thermoregulation will impose a fitness cost or decrement. Regardless of whether distribution of individuals on a thermal gradient is due to underlying variation in preferred temperature or the transient occupation of different temperatures within a range of preferred or non-lethal temperatures, deviations from the optimal fitness temperature should lower population mean fitness, generating a "performance load" on the population (*sensu *[28,29]). Thus a corollary to the thermal coadaptation hypothesis is that natural selection should act to decrease the performance load of a population under environmental conditions in which variation in performance has its largest effect on overall fitness.

A complication in testing the relationship between thermal preference and Darwinian fitness is that the appropriate measure of fitness differs depending on the specific demographic context of the population involved. Fitness is commonly estimated either as reproductive output, *R*_o _[or its individual level equivalent Lifetime Reproductive Success (LRS), the total number of offspring produced during a female's lifetime] or as the intrinsic rate of population increase (*r*), which is influenced both by offspring number and reproductive timing. Because *r *is strongly and inversely related to generation time [30], which decreases with temperature in ectotherms, temperatures that maximize *r *are generally higher than those that maximize rate-insensitive fitness metrics *R*_o _and LRS (Figure 1B) [3]. Thus, thermal preference behaviors can maximize *R*_o _(or LRS) or *r*, but not both simultaneously (Figure 1B). The question then becomes which measure of fitness should correspond with thermal preference? Evolutionary ecologists [31-35] have suggested that the relevant fitness measure can be identified using basic demographic information (*e.g*. knowing whether the population is stable or expanding, or has discrete or overlapping generations). However, these factors are frequently unknown for natural populations, making *a priori *identification of the appropriate fitness metric impossible.

Here we study thermal coadaptation in *Caenorhabditis elegans*. These nematodes are very small (~1 mm as adults) and are easily maintained in the laboratory [36]. Consequently, the distribution and *T_p _*of large numbers of individuals can be determined on controlled thermal gradients. Moreover, fitness (*e.g*. [37-41]) - and specifically the thermal dependence of fitness [42-44] - can also be repeatedly and reliably estimated in *C. elegans*. Although the demographic status of these worms is unknown, both LRS and *r *are readily estimable in this system: thus we can examine the relationship between thermal preference and both fitness metrics. Accordingly, we determined the *T_p _*and the thermal dependence of both fitness measures for eight natural isolates of *C. elegans *and then explored whether *T_p _*corresponds with temperatures that maximize fitness.

## Methods

### Thermal preference and fitness assays

Natural isolates of *Caenorhabditis elegans *(N = 8, Table 1) were obtained from the *Caenorhabditis *Genetics Center (University of Minnesota, Minneapolis, MN), inbred for 10 generations, and immediately frozen to minimize further genetic changes. Stocks of these inbred lines were allowed to recover from freezing for at least two generations under standard maintenance conditions, on Nematode Growth Medium-lite (NGM-lite, US Biological, Texas, USA) seeded with *Escherichia coli *strain OP50 at 20°C, prior to thermal preference and fitness assays. We excluded the N2 strain from our analysis because it shows signs of behavioral adaptation to laboratory conditions [45-48], but we present fitness results for N2 in Additional file 1: Fig. S1.

**Table 1:** Geographic origin of *C.elegans *natural isolates used in this study.

Strain	Origin
AB3	Adelaide, Australia
CB4854	Altadena, CA, USA
CB4855	Palo Alto, CA, USA
CB4856	Hawaii, USA
CB4857	Claremont, CA, USA
JU258	Fibeiro Frio, Madeira
JU262	Le Blanc, France
PX174	__*

*Based on genomic information [54] PX174 appears to be a lab derived sub-isolate of RC301, which is from Freiburg, Germany.

Thermal preference was assayed as previously described [45]. Briefly, age-synchronized populations of final larval stage worms (L4), reared at 20°C, were applied to thermal gels [NGM-lite in plastic frames (10.2 × 17.5 × 0.5 cm) seeded with *E. coli *strain OP50] on stable linear thermal gradients (10.5°C - 29°C) at a starting point corresponding to 24°C. The positions of individual worms were scored after one hour. The temperature chosen by each individual was then estimated from its position on the thermal gradient using a linear model that relates gradient position to temperature. Results were analyzed using analysis of variance (ANOVA) with isolate as the main effect and replicate as a random nested effect. Standard errors were calculated from the model, with replicate as the unit of sampling variation, using a least-square means approach. Differences in thermal preferences among isolates were evaluated using Tukey's HSD, α = 0.05. All analyses were performed in JMP 4.0 (SAS Institute, Cary, NC, USA).

Fitness (LRS and *r*) was assayed at seven temperatures (10°C, 12°C, 14°C, 15°C, 20°C, 23°C, and 25°C) in four isolates (CB4854, CB4856, CB4857, PX174) with extreme thermal preferences. Subpopulations of each isolate were allowed to acclimate to the assay temperature for at least two generations prior to use in fitness assays. Individual L4 hermaphrodites, reared from eggs laid by 30 adults within a 3-h period, were transferred to individual Petri dishes (35 × 15 mm) containing NGM-lite seeded with OP50 and incubated at the assay temperature. Reproductively active individuals were subsequently transferred to new dishes at 24-hour intervals. At the highest temperatures worms were transferred more frequently to prevent offspring overcrowding. Offspring produced during each 24-h period were counted at maturity. LRS was calculated as total lifetime fecundity at each temperature and *r *was calculated from, *∑e^-rx^l_x_m_x _*= 1 where *l_x _*is age specific survivorship to day *x*, and *m_x _*is the fecundity at day *x *[39]. Results were analyzed using a full factorial ANOVA with isolate and temperature as main effects (data from 25°C were excluded from this analysis because PX174 did not reproduce at this temperature). The isolate-specific effects of temperature on fitness were analyzed using ANOVA with isolate as the main effect at each temperature. Differences among isolates were evaluated using LSMeans Contrasts and Tukey's HSD.

### Correspondence between thermal preference and fitness

To determine the correspondence between preference and the two fitness metrics, each isolate's preference for a given temperature, as indicated by the fraction of worms positioned at that temperature (10°C, 12°C, 14°C, 15°C, 20°C, 23°C, and 25°C; ± 0.5) on the thermal gradient was correlated with the fitness estimate associated with each of the seven assayed temperatures. If thermoregulation is effective, then the fraction of worms found at a given temperature will correlate positively and strongly with the fitness at that temperature. This approach takes advantage of information from the shape of each isolate's thermal preference distribution and fitness performance curve (*i.e*. the convolution of the distributions). Note that this is a within-isolate preference-performance measure, as opposed to a between-isolate measure, such as the correlation between *T*_p _and *T_max _*among isolates.

### Consequences of thermal preference on average fitness

To determine the average expected fitness (*w_exp_*) of worms in a thermal gradient for each isolate, we estimated fitness at temperatures from 9°C - 29°C via linear interpolation of measured values and integrated these data over the isolate-specific thermal preference frequency distribution. The integral was approximated by summing the product of the temperature-specific fitness measures and the proportion of total individuals displaying a preference to that temperature. To quantify the deficit in fitness between the maximum fitness possible (*w_max_*, if individuals thermoregulate perfectly) and the expected average fitness (based on the actual distribution of *T*_p_), we calculated the Performance Load as *PL = (w_max _- w_exp_)/w_max_*. *PL *measures the proportional reduction in fitness of the average individual in a isolate given the thermal preference distribution of that isolate relative to the maximum observed fitness. *PL *ranges from 0 to 1 where 0 means that all individuals achieve equal, thus maximum, fitness and 1 means that all but one individual has zero fitness.

## Results

### Thermal preferences in *C. elegans *natural isolates

**Figure 2:**
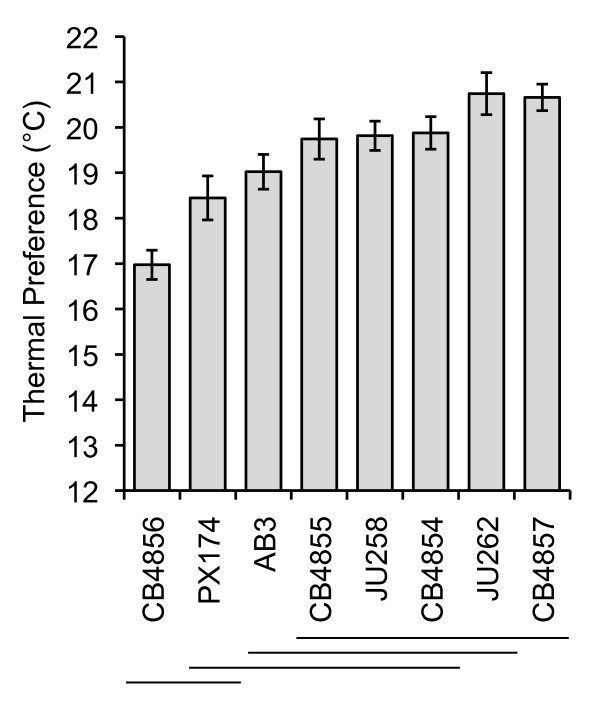
**Variation in mean thermal preference among a global sample of *C. elegans *natural isolates**. Mean thermal preferences are based on an average of 4 replicates and 233 individuals per replicate, 7484 total individuals. Values are least square means ± SEM. Isolates not connected by the same horizontal line are significantly different from one another (Tukey's HSD, *p *≤ 0.05).

We find that *T_p _*varies significantly among natural isolates of *C. elegans *(*F*_7,23.87 _= 13.22, *p *< 0.0001), with *T_p _*ranging from 16.9 ± 0.3 to 20.7 ± 0.5°C (mean ± SEM) (Figure 2). Variation among replicates within isolates is also significant (*F*_24,7452 _= 6.52, *p *< 0.001). However, this among-replicate variance accounts for only 2.4% of the total variation, suggesting that the significant among-replicate variation likely reflects the large number of individuals sampled within each replicate (average of 233; 7484 total).

**Figure 3:**
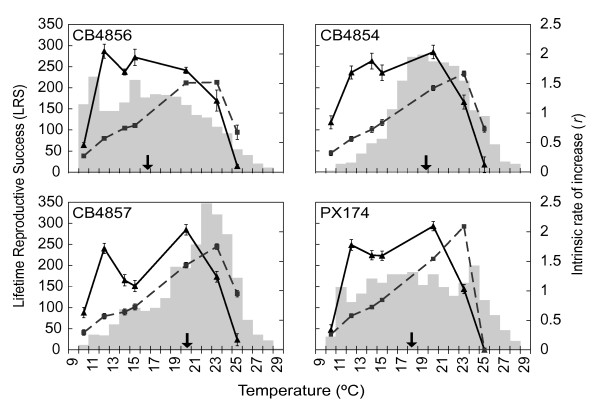
**Thermal sensitivity of two measures of fitness, Lifetime Reproductive Success (LRS, solid lines) and intrinsic rate of increase (*r*, dashed lines), and the relative distribution of preferred temperatures (grey histograms)**. *r *increases with temperature to about 23°C before rapidly declining. LRS is relatively high over a relatively broad range of temperatures. Fitness values are least square means ± SEM, and mean LRS and *r *at each temperature are calculated from an average of 15 individuals per strain at each temperature, 402 total individuals. Each thermal preference distribution is based on the responses of an average of 1090 individuals. Arrows indicate mean thermal preferences.

The relative frequency distributions of *T_p _*for CB4854, CB4856, CB4857, and PX174, the four isolates for which temperature dependent fitness was also determined, are illustrated in Figure 3 (gray bars). Each isolate has a characteristic *T_p _*distribution across the gradient, with CB4856 tending toward cold temperatures, CB4854 and CB4857 tending toward progressively hotter temperatures, and PX174 dispersing nearly uniformly across most of the gradient but still avoiding the extremes.

### Temperature dependent fitness

The thermal sensitivities of LRS and *r *vary by isolate (effect of isolate: LRS *F_3, 341 _*= 6.63, *p *= 0.0002; *r F_3, 341 _*= 27.23, *p *< 0.0001; isolate x temperature interaction: LRS *F_15, 341 _*= 4.97, *p *< 0.0001; *r F_15, 341 _*= 18.36, *p *< 0.0001; see also Additional file 2: Fig. S2 and Additional file 3: Fig. S3) but nonetheless show some similarities among isolates. Generally, *r *increases with temperature up to about 23°C before declining steeply for all isolates (Figure 3). LRS increases sharply from 10°C to 12°C, remains relatively high between 12°C and 20°C (except in CB4857), but then declines rapidly at higher temperatures (Figure 3). CB4857 is unique in that it produces fewer offspring at 14°C and 15°C than all other isolates (LSMeans Contrast: 14°C *F_1, 62 _*= 19.00, *p *< 0.001; 15°C *F_1, 45 _*= 13.53, *p *< 0.001). Overall, *C. elegans *natural isolates are able to maximize LRS, but not *r*, over a broad range of temperatures (Figure 3). In other words, *r *is more thermally sensitive than is LRS.

### Coadaptation

**Figure 4:**
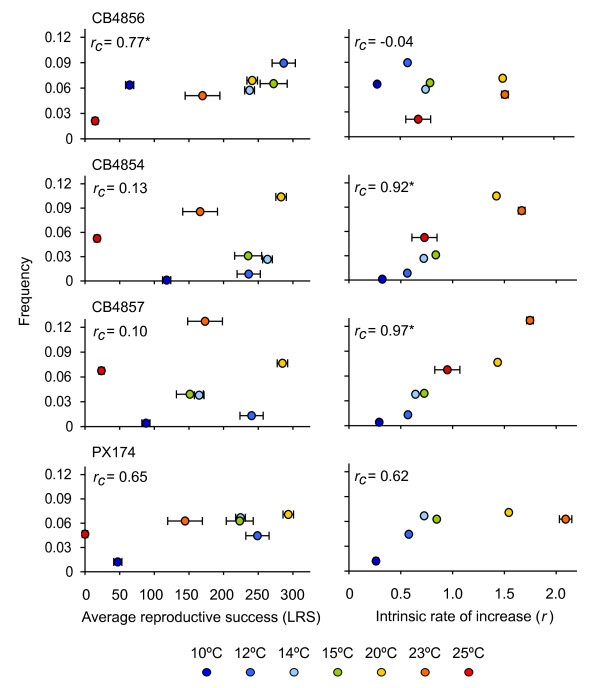
**Correspondence between thermal preference and different fitness measures**. In CB4856, thermal preference (the frequency of worms found at each temperature) correlates with LRS (left panel) but not *r *(right panel). The reverse is true for both CB4854 and CB4857, in which thermal preference correlates strongly with *r*. In PX174 thermal preference is correlated positively and about equally with both fitness measures, due to its broad, flat frequency distribution on a thermal gradient. Fitness values are least square means ± SEM. *p < 0.05.

**Figure 5:**
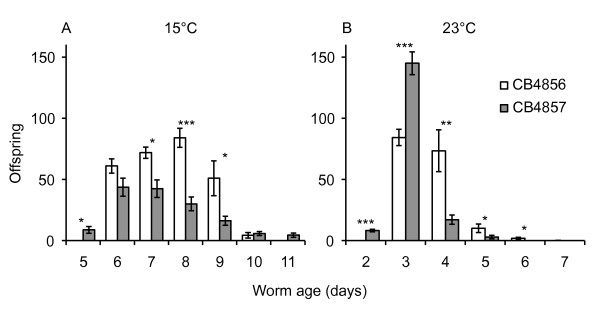
**Age and temperature specific offspring production in natural isolates CB4856 and CB4857**. A) The cold-seeking isolate CB4856 produces more offspring than the hot-seeking isolate CB4857 at 15°C (*F_1, 17 _*= 13.42, *p *= 0.0019). B) In contrast, at 23°C, LRS does not differ between CB4856 and CB4857 (*F_1, 23 _*= 14.52, *p *= 0.8779), but CB4857 produces significantly more offspring than CB4856 at ages 2 and 3 days which positively impacts *r*. Differences between strains at each age were analyzed using ANOVA with strain as the main effect. *p < 0.05, **p < 0.001, ***p < 0.0001.

We find that isolates showing marked thermal preference (CB4854, CB4856, CB4847; Figure 3) do prefer temperatures that maximize fitness; however, the aspects of fitness maximized differ depending on whether the isolates have cool versus warm thermal preferences. In CB4856, the isolate with the coolest *T_p _*(Figure 2), few worms are found at 25°C (Figure 4A, red circle), a relatively warm temperature at which its reproductive output is low. CB4856 worms more frequently prefer lower temperatures that are associated with higher reproductive output (LRS). Consequently, the correlation between thermal preference and LRS in CB4856 is much higher (*r_c _*= 0.77, *p *= 0.04) than that between preference and *r *(*r_c _*= -0.04, *p *= 0.93; Figure 4B). In contrast, in two isolates with relatively warm *T_p _*(Figure 2), CB4854 and CB4857, thermal preference is correlated tightly with *r *(*r_c _*= 0.92, *p *< 0.01 and *r_c _*= 0.97, *p *< 0.01 respectively) but not LRS (*r_c _*= 0.13 and *r_c _*= 0.10, p > 0.01). In PX174, LRS and *r *show nearly identical, lower correlations with preference (LRS, *r_c _*= 0.65, *p *= 0.12; *r*, *r_c _*= 0.62, *p *= 0.14) likely due to the absence of marked thermal preference behavior in this isolate (*i.e*. the thermal preference distribution for this isolate is relatively flat). Differential fitness effects also influence correlations between fitness and preference (Figure 5).

### Performance cost of imperfect thermoregulation

The failure of organisms to narrowly prefer and occupy fitness-optimizing temperatures generates a potential "performance load" on mean population fitness, which we calculate here by summing the preference distributions over the thermal fitness curves (thus the load measures the average proportional reduction in fitness relative to the optimum on a scale from 0 to 1). Overall, performance loads are moderate to large (0.25 - 0.37 for LRS and 0.28 - 0.50 for *r*; Table 2). For example, PX174 achieves only 50% of its potential maximal fitness (*r*) because of sub-optimal thermoregulatory behavior.

**Table 2:** Average expected fitness for each isolate calculated by integrating temperature-dependent fitness measures over the observed thermal preference distributions ("Obs.") compared to the maximum fitness expected is all individuals chose the single best temperature ("Max.")

	LRS			***r***		
	
Isolate	Obs.	Max.	***PL***	Obs.	Max.	***PL***
CB4857	178	285	0.37	1.25	1.75	0.29
PX174	192	293	0.34	1.04	2.09	0.50
CB4856	211	287	0.26	0.95	1.52	0.38
CB4854	213	285	0.25	1.20	1.67	0.28

The relative difference between these yields the Performance Load (*PL*).

## Discussion

Many ectotherms use behavioral adjustments (*e.g*. in habitat choice, posture, and timing of activity) to regulate body temperatures within preferred ranges [8,12,49-52] thereby avoiding damaging and lethal temperatures as well as maximizing time spent at or near physiologically optimal temperatures. The classical co-adaptational hypothesis [12,18,21] predicts that thermal preferences should be centered at or near temperatures that maximize fitness (but see [6]). Moreover, evolutionary shifts in thermal preferences should drive compensatory shifts in the thermal sensitivity of fitness and *vice versa *[18,21]. In this study, we assayed thermal preference and Darwinian fitness in natural isolates of *C. elegans *to test this coadaptational hypothesis.

### Coadaptation

Does thermoregulatory behavior maximize fitness in the isolates of *C. elegans *studied here? We find that preferred temperatures do correspond with temperatures maximizing fitness, but that the relationship between thermal preference and different aspects of fitness varies among natural isolates of *C. elegans *(Figures 3, 4). Intraspecific variation in thermal preference-fitness relationships is not predicted by the classical preference-fitness coadaptation hypothesis. Whereas the coadaptation hypothesis predicts that differences in *T_p _*among populations should be accompanied by corresponding shifts in *T_max _*(Figure 1A), in our data, fitness curves are not systematically shifted such that *T_p _*consistently corresponds with *T*_max _of either *r *or LRS. Rather, the isolate with the coolest *T_p _*favors LRS, while the two isolates preferring a warmer *T_p _*(CB4854 and CB4857) favor *r*. Under these circumstances it is not surprising that isolates with high *T*_p _maximize *r *rather than LRS and *vice versa *due to the negative relationship between generation time and temperature (Figure 1B) [3,7]. We find that correlations between fitness and thermal preference are driven primarily by isolate-specific differences in thermal preference, but the effects of temperature on fitness also vary among isolates. This is most clearly seen in temperature-dependent reproductive rates among isolates, in which cold-seeking CB4856 strongly outperforms hot-seeking CB4857 at cold temperatures and *vice versa *(Figure 5). Some of the differentiation among isolates may be facilitated by the largely self-fertilizing mode of reproduction in *C. elegans*, which tends to increase linkage disequilibrium across the genome [53,54]. There are obviously too few isolates here to definitely define new temperature-fitness rules within this species, but these data strongly suggest that preference-fitness relationships are more complicated than predicted by the classical coadaptation hypothesis.

The optimization of different aspects of fitness under different ecological and/or demographic conditions is predicted by life history theory [32,33,35]. Specifically, total offspring production, *R_o _*or LRS, should be optimized in stable populations where population size is regulated by density-dependent factors, whereas the intrinsic rate of increase, *r*, should be optimized in density-independent populations where natural selection should favor early reproduction [32,33,35,55]. Therefore, if the coadaptation hypothesis is indeed correct, different components of fitness (*e.g*. total reproduction versus rate of reproduction) might have been under selection in different isolates of *C. elegans*. The specific structure of these predictions is likely to benefit from more detailed models of the relationship between metabolism and population dynamics (*e.g*. [5]).

It is almost certainly true, however, that the evolution of thermal preference-fitness relationships is also influenced by interacting genetic, genomic, physiological, ecological, and environmental factors. For example, important life history traits and genetic correlations between these traits (*e.g*. age at maturity, egg number, and egg size, and the genetic control of phenotypic plasticity) are temperature-dependent in *C. elegans *[43,56-58]. Moreover, soil nematodes live in highly dynamic thermal environments [59-61]; and nematode community structure is both complex [59] and strongly influenced by temperature [62-65]. Additionally, the impact of temperature on performance may differ depending on physiological context and resource availability [66]. In any case, the fact that Performance Loads are fairly high in all *C. elegans *isolates (Table 2) suggests that *T_p _*is not strongly optimized in this species, and possibly reflects the effects of complex ecological, environmental, and genetic interactions on the evolution of thermal preference-fitness relationships. Thus, while we are able to robustly measure the effect of temperature of various components of fitness in the laboratory environment, true fitness in nature awaits measurement in the field.

### Fitness

Although the effects of temperature on fitness vary among isolates of *C. elegans *(Figures 3, S2, S3) (see also [44,67]), this species can generally be described as a "*Jack of all temperatures*" [68] in terms of LRS. The isolates studied here produce large numbers of offspring (≥ 75% of maximal production) over up to 64% (CB4854) of their reproductive temperature range (9°C to 26°C, herein). *Caenorhabditis briggsae*, an androdioecious sister species to *C. elegans*, has similarly high offspring production (≥ 75% of maximal production) over 63% of its reproductive temperature range [69]. However, *C. briggsae *reproduces at a warmer range of temperatures than *C. elegans *(14°C to 30°C; [69]).

The thermal sensitivity of reproductive output in *C. briggsae *appears to vary with latitude; isolates of tropical origin produce more offspring at high temperatures than do isolates from temperate regions [69]. This pattern is not evident in *C. elegans*; the lowest latitude isolate we studied (CB4856) does not produce more offspring at high temperatures than do other isolates. There is some evidence, however, that thermal preference varies with latitude in *C. elegans *(*r^2 ^*= 0.60, p = 0.11); this suggestive result is entirely driven by CB4856. To date, too few isolates of *C. elegans *and of *C. brigg*sae from geographically diverse, well-characterized collections (*i.e*. known microclimate and elevation) have been characterized for both thermal preference and temperature dependent fitness to define relationships among thermal preference, fitness, and latitude, or other ecologically relevant clines.

### Behavioral variation

Thermal preference behavior has been studied in *C. elegans *for more than 30 years [70] and has led to advances in the fields of genetics [71-76], neuroscience [77-80], and the study of memory and learning [74,78,81]. Even so, findings from many of these studies are somewhat problematic because seemingly conflicting results have been obtained by different laboratories, apparently driven in part by sensitivity to environmental conditions such as nutritional state and cultivation temperature (*e.g*. [45,74,82]). Thermal gradient steepness can also influence thermal preference behavior in some isolates [83]. The standard laboratory strain N2 only displays typical thermal preference behaviors on gradients < 1°C/cm, whereas natural isolates of *C. elegans*, such as those assayed here, are less sensitive to gradient steepness [83]. Here, we assayed thermal preference in eight isolates of *C. elegans*, under identical experimental conditions. Thus, our data clearly illustrate that thermal preference behavior varies significantly among natural isolates in *C. elegans *(see also [45,83]). Environment-dependent and isolate-specific variation in thermal preference behavior might be informative from an evolutionary perspective because a behavior with such important effects on fitness might be fine tuned to be sensitive to a number of possible environmental cues. In fact, it would be surprising if we did not find variation in this important behavior given the large potential fitness effects of thermal preference behavior and the fact that *C. elegans *populations are found around the world in different climates. Using genetic differentiation among natural isolates as means of probing this system [84] should help to distinguish evolutionary signal from noise [45,83]. However, our results show that interpretation of natural variation, and probably any variation in this trait, must be performed in the context of the ecological causes and consequences of that variation.

## Conclusions

Most studies of the coadaptation of thermal preference and fitness have examined only performance traits and not Darwinian fitness *per se*. Here we show that thermal preferences can indeed correspond with temperatures maximizing Darwinian fitness in *C. elegans*, but not as predicted by the coadaptation hypothesis. We find that the relationship between thermal preference and fitness varies among isolates such that isolates that prefer relatively warm temperatures, CB4854 and CB4857, maximize the timing of offspring production (*r*) and the isolate that prefers a cooler temperature, CB4856, maximizes offspring number (LRS). Thus, we demonstrate that preference-fitness relationships can be more complex that predicted by current theory. Our results also demonstrate that the choice of fitness measure can have a large effect on the interpretation of the coadaptedness of thermal preference and fitness: had we measured only r, for instance, we would have concluded that thermal preferences were not correlated with fitness. Finally, despite the tight correlations between thermal preference and specific measures of fitness, the isolates examined here still demonstrate substantial performance loads caused by imprecision of individual preference behavior. Coupling this individual variation with other known external environmental drivers such as rearing temperature (*e.g*. [70,83]) and potential unknown temperature correlates such as predation risk and inter-specific competition suggests that co-adaptation of *T*_p _and thermal sensitivity of fitness is likely to depend on overall interactions involving ecology, environmental experience, behavior and fitness.

## Authors' contributions

Study designed by PCP, RBH, LA, and JLA. Experiments performed by LA, JLA, and BE. Data analyzed by JLA. The manuscript was written by JLA, LA, RBH, and PCP. All authors read and approved the final manuscript.

## Supplementary Material

Additional file 1 **Fig. S1**. Temperature-dependent fitness response in the standard lab strain N2. Click here for file

Additional file 2 **Fig. S2**. Temperature dependence of Lifetime Reproductive Success (LRS) in four strains of *C. elegans*. Click here for file

Additional file 3 **Fig. S3**. Temperature dependence of the intrinsic rate of increase (*r*) in four strains of *C. elegans*. Click here for file
