# Supplementary material for: Does thermoregulatory behavior maximize reproductive fitness of natural isolates of Caenorhabditis elegans?
Source: BMC Evol Biol. 2011 Jun 6;11:157. doi: 10.1186/1471-2148-11-157 (PMC3141425; doi:10.1186/1471-2148-11-157)
Supplement: Additional file 1 — Fig. S1. Temperature-dependent fitness response in the standard lab strain N2. [file 1471-2148-11-157-S1.PDF]

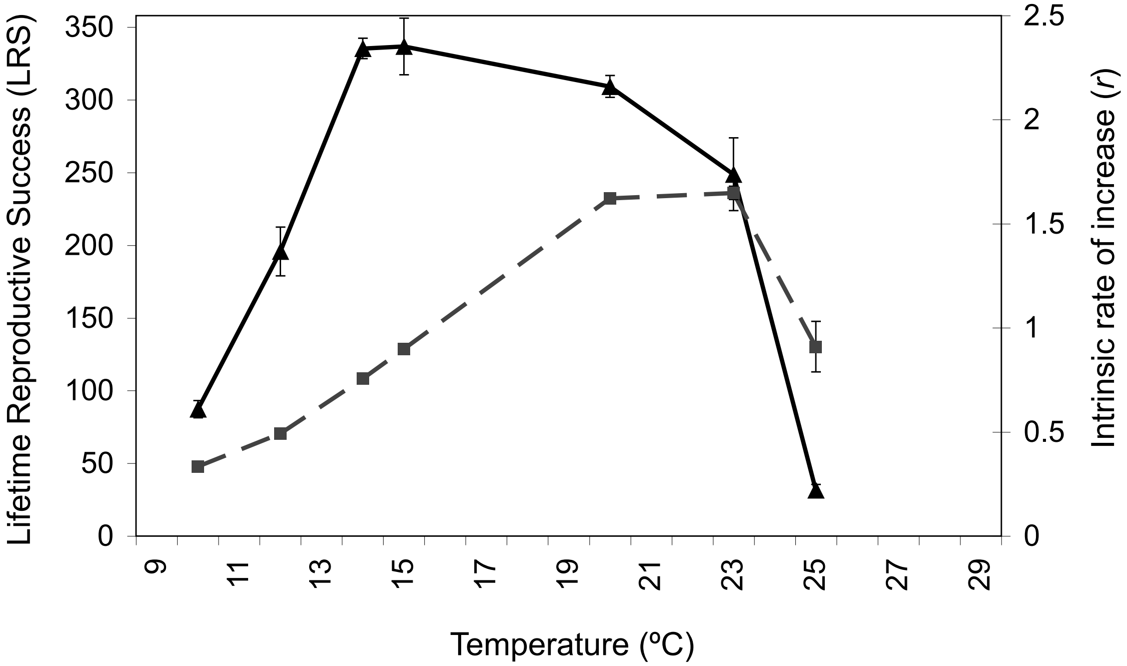

Fig. S1. Temperature-dependent fitness response in the standard lab strain N2. The solid line shows lifetime reproductive success and the dashed line shows the intrinsic rate of increase.
