# Supplementary material for: Does thermoregulatory behavior maximize reproductive fitness of natural isolates of Caenorhabditis elegans?
Source: BMC Evol Biol. 2011 Jun 6;11:157. doi: 10.1186/1471-2148-11-157 (PMC3141425; doi:10.1186/1471-2148-11-157)
Supplement: Additional file 2 — Fig. S2. Temperature dependence of Lifetime Reproductive Success (LRS) in four strains of C. elegans. [file 1471-2148-11-157-S2.PDF]

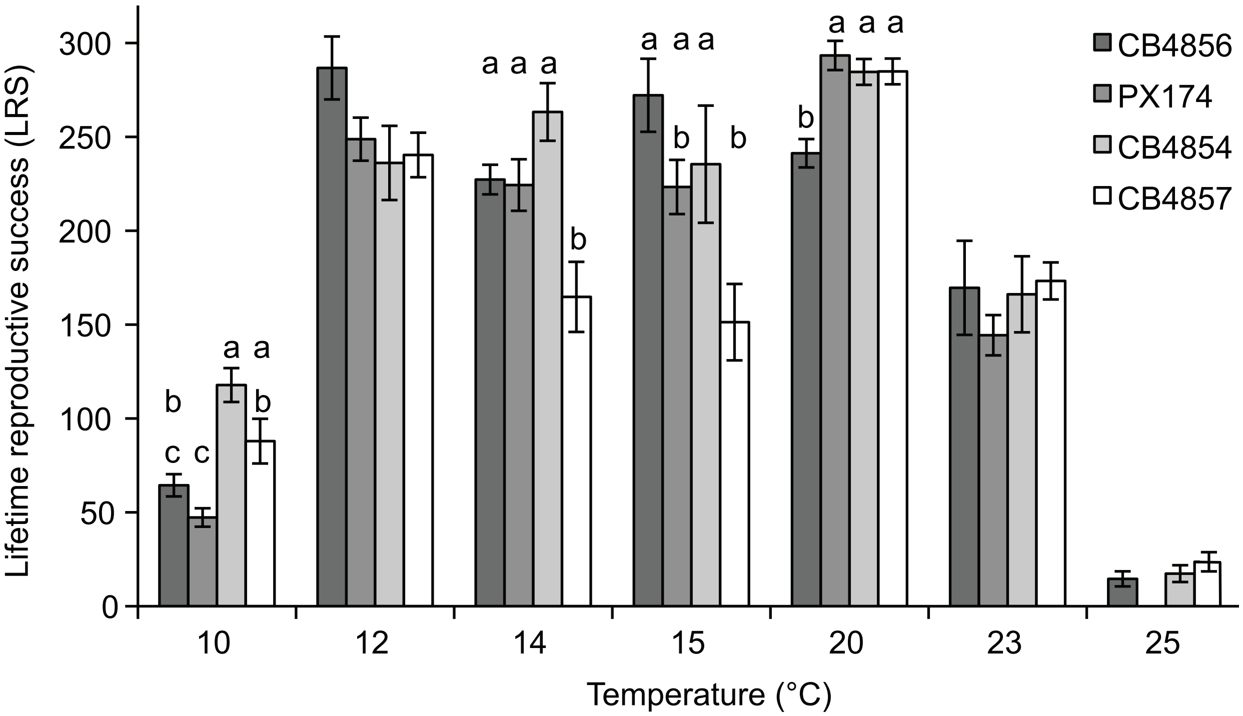

Fig S2. Temperature dependence of lifetime Reproductive Success (LRS) in four strains of *C. elegans*. Values are least square means ± 1 s.e.m. Results were analyzed for each temperature using analysis of variance with strain as the main effect. 10°C: $F_{3, 60} = 12.93$, $p < 0.001$. 12°C: $F_{3, 49} = 1.37$, $p = 0.26$. 14°C: $F_{3, 62} = 7.07$, $p < 0.001$. 15°C: $F_{3, 45} = 4.60$, $p = 0.007$. 20°C: $F_{3, 70} = 11.58$, $p < 0.001$. 23°C: $F_{3, 55} = 0.84$, $p = 0.48$. 25°C: $F_{2, 34} = 1.09$, $p = 0.35$. Comparisons among strains were performed using Tukey's HSD. At each temperature, strains not connected by the same letter are significantly different (Tukey's HSD $\alpha = 0.05$). Letters are not reported in cases where significant differences were not observed.
