# Supplementary material for: Does thermoregulatory behavior maximize reproductive fitness of natural isolates of Caenorhabditis elegans?
Source: BMC Evol Biol. 2011 Jun 6;11:157. doi: 10.1186/1471-2148-11-157 (PMC3141425; doi:10.1186/1471-2148-11-157)
Supplement: Additional file 3 — Fig. S3. Temperature dependence of the intrinsic rate of increase (r) in four strains of C. elegans. [file 1471-2148-11-157-S3.PDF]

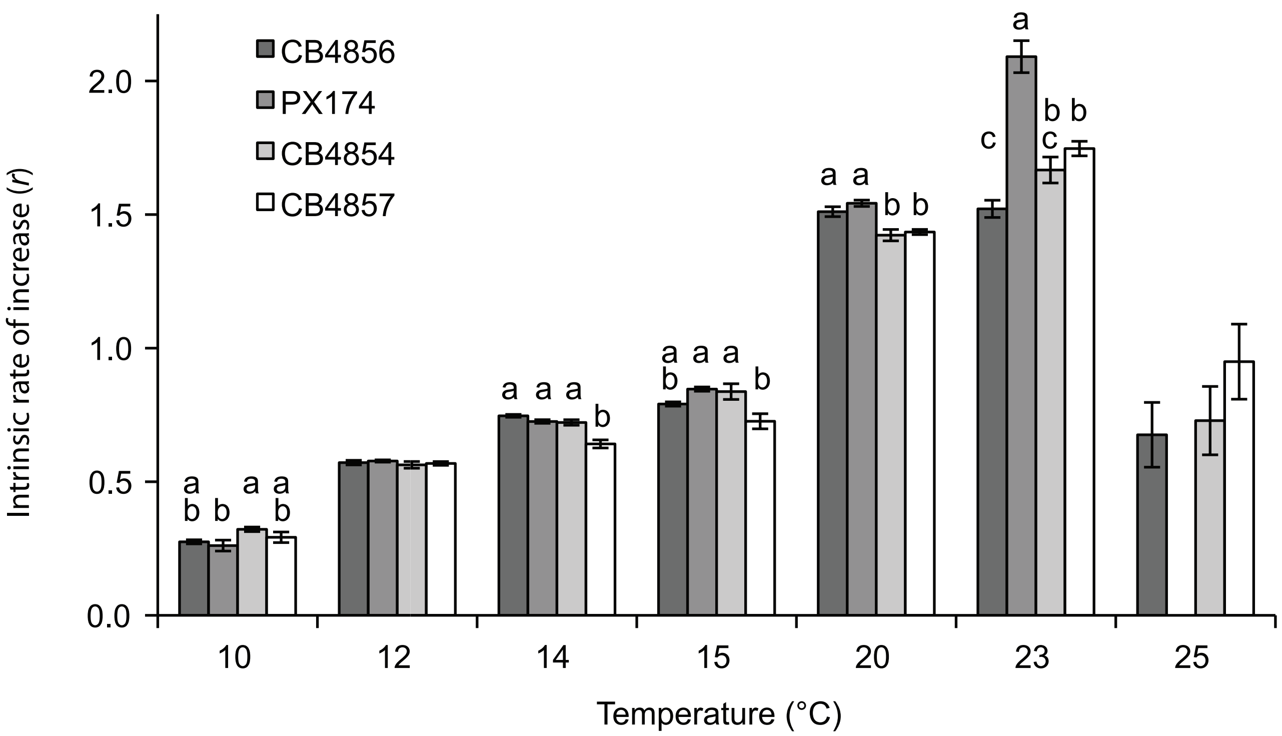

Fig S3. Temperature dependence of the Intrinsic rate of increase (*r*) in four strains of *C. elegans*. Values are least square means ± 1 s.e.m. Results were analyzed for each temperature using analysis of variance with strain as the main effect. 10°C: $F_{3, 60}$ = 3.00, p = 0.037. 12°C: $F_{3, 49}$ = 0.47, p = 0.71. 14°C: $F_{3, 62}$ = 25.26, p < 0.001. 15°C: $F_{3, 45}$ = 7.79, p < 0.001. 20°C: $F_{3, 70}$ = 11.06, p < 0.001. 23°C: $F_{3, 55}$ = 24.77, p < 0.001. 25°C: $F_{2, 34}$ = 1.26, p = 0.30. Comparisons among strains were performed using Tukey's HSD. At each temperature, strains not connected by the same letter are significantly different (Tukey's HSD α = 0.05). Letters are not reported in cases where significant differences were not observed.
